# High phosphate diet suppresses lipogenesis in white adipose tissue

**DOI:** 10.3164/jcbn.17-141

**Published:** 2018-07-11

**Authors:** Yukiko Imi, Norie Yabiki, Maerjianghan Abuduli, Masashi Masuda, Hisami Yamanaka-Okumura, Yutaka Taketani

**Affiliations:** 1 Department of Clinical Nutrition and Food Management, Institute of Biomedical Sciences, Tokushima University Graduate School, 3-18-15 Kuramoto-cho, Tokushima 770-8503, Japan

**Keywords:** dietary phosphate, white adipose tissue, insulin, lipogenesis, lipolysis

## Abstract

Excessive phosphate intake has been positively associated with renal and vascular dysfunction, conversely negatively associated with body fat accumulation. We investigated the effect of a high-phosphate diet on the expression of lipid metabolic genes in white adipose tissue and liver. Male 8-week-old Sprague–Dawley rats were fed a control diet containing 0.6% phosphate or a high-phosphate diet containing 1.5% phosphate for 4 weeks. In comparison to the control group, the HP group showed a significantly lower body fat mass and fasting plasma insulin level alongside decreased lipogenic and increased lipolytic gene expression in visceral fat. Additionally, the expression of genes involved in hepatic lipogenesis, hepatic glycogenesis, and triglyceride accumulation decreased in the high-phosphate group. Exogenous phosphate, parathyroid hormone, and fibroblast growth factor 23 did not directly affect the expression of lipolytic or lipogenic genes in 3T3-L1 adipocytes and HepG2 hepatocytes. Thus, the high-phosphate diet suppressed the activity of white adipose tissue by increasing lipolytic gene expression and decreasing lipogenic gene expression. These effects could have been caused by the lowered fasting plasma insulin level that occurred in response to the high-phosphate diet, but were not directly caused by the increases in plasma phosphate or phosphaturic hormones.

## Introduction

Inorganic phosphate is an essential nutrient in biological processes including bone formation, the biosynthesis of phospholipids and nucleic acids, intracellular signaling, and energy metabolism.^(1)^ For those processes to function correctly, the concentration of phosphate in serum must be maintained within a certain range by bone formation/resorption, intestinal absorption, and renal reabsorption.^(2,3)^ Since renal reabsorption plays a critical role in the maintenance of serum phosphate levels in mammals, hyperphosphatemia is a general symptom in chronic kidney disease (CKD) patients. Hyperphosphatemia causes vascular calcification and endothelial dysfunction, eventually resulting in cardiovascular events including heart failure and myocardiac infarction.^(4,5)^ Recent studies have demonstrated that high serum phosphate levels or an excessive intake of phosphate are positively associated with the morbidity and mortality of cardiovascular disease in CKD patients and in the general population.^(6,7)^

By contrast, several epidemiological studies have demonstrated a negative relationship between serum phosphate levels and the risk of being overweight or having metabolic syndrome.^(8–10)^ Furthermore, a recent human study demonstrated that the daily amount of dietary phosphate intake showed a negative correlation with body mass index.^(11)^ Additionally, an animal study demonstrated that a low dietary intake of phosphate by apolipoprotein E-deficient mice induced metabolic disorders including fat accumulation, liver steatosis, and insulin resistance.^(12)^ Conversely, a high-phosphate diet suppressed weight gain and visceral adipocyte hypertrophy in uninephrectomized db/db mice.^(13)^ Concordantly, dietary phosphorus supplementation prevented weight gain and reduced waist circumference in overweight and obese adult humans.^(14)^ Furthermore, our recent study showed that a high-phosphate diet resulted in a reduction of visceral fat in healthy Sprague–Dawley rats.^(15)^

Physiological phosphate homeostasis is regulated by several hormones including 1,25-dihydroxyvitamin D [1,25(OH)_2_D], parathyroid hormone (PTH), and fibroblast growth factor 23 (FGF23).^(16)^ When the concentration of phosphate in serum increases, PTH and FGF23 are secreted and suppress renal phosphate reabsorption. Therefore, an excessive dietary intake of phosphate induces the secretion of PTH and FGF23.^(17,18)^ Several studies have suggested that elevated PTH levels may be a risk factor for metabolic syndrome.^(19,20)^ In adipocytes, PTH induced insulin resistance and fatty acid release.^(21)^ FGF23 also affected obesity in people with normal kidney function.^(22)^

These studies suggest that daily phosphate intake and the concentration of phosphate or phosphate-regulating hormones in serum may influence systemic lipid and glucose metabolism. Recently, a DNA microarray analysis showed that a high-phosphate diet affected lipid metabolism in rat liver.^(23)^ However, the effect of excess phosphate intake on white adipose tissue has not been clarified yet. A recent study showed that a high concentration of extracellular phosphate activated the Akt/mTORC pathway in 3T3-L1 adipocytes.^(24)^ In the present study, we focused on the changes of lipid metabolism in the white adipose tissue of healthy rats fed a high-phosphate diet.

## Materials and Methods

### Animal study

Male 8-week-old Sprague–Dawley rats (Japan SLC, Inc., Shizuoka, Japan) were individually caged in a climate-controlled room (23 ± 1°C) with a 12-h light/dark cycle. The rats were randomly divided into two groups and fed a control (CP) diet containing 0.6% phosphate and 0.6% calcium or a high-phosphate (HP) diet containing 1.5% phosphate and 0.6% calcium. The experimental diets were based on the standard AIN-93G diet and their detailed compositions are shown in Table 1. The rats were fed these diets for 4 weeks using the pair-feeding procedure and were granted free access to deionized water. After 4 weeks, all the rats were deprived of food for 12 h and euthanized under anesthesia. Blood samples were collected into a tube containing heparin and centrifuged to obtain plasma. Plasma and vesical urine were stored at –20°C until further analysis. Liver and fat were harvested and stored at –80°C. The experiment was performed in accordance with the guidelines for the care and use of animals at Tokushima University.

### Biochemical analysis

The blood glucose level was determined using the Accu-Chek blood glucose meter (Roche Diagnostics, Basel, Switzerland) before euthanasia. The concentrations of phosphate, calcium, and creatinine in the plasma and urine were measured using the phospha-C, calcium-E, and creatinine tests (Wako Pure Chemical Industries, Osaka, Japan), respectively. Plasma triglyceride, cholesterol, and non-esterified fatty acid levels were measured using the triglyceride-E, cholesterol-E, and NEFA-C tests (Wako Pure Chemical Industries), respectively. The concentrations of PTH and FGF23 in plasma were measured using a rat intact PTH ELISA kit (Immutopics, San Clemente, CA) and an FGF23 ELISA Kit (Kinos, Tokyo, Japan). Plasma insulin concentrations were measured using an ultra-sensitive rat insulin ELISA kit (Morinaga, Kanagawa, Japan). The concentrations of 1,25(OH)_2_D in plasma were measured using the radioimmunoassay 2-antibody method (SRL, Tokyo, Japan).

### Measurement of hepatic and fecal triglyceride

 Hepatic and fecal triglyceride were extracted using chloroform/methanol (2:1) in accordance with the method of Folch *et al.*^(25)^ The extracts were used for triglyceride analysis by the same method as used for the analysis of plasma samples. Hepatic triglyceride contents were normalized by liver weight. The amount of fecal triglyceride excretion were normalized fecal weight per day.

### Cell culture

The 3T3-L1 pre-adipocytes were kindly provided by Dr. T. Hosaka (Kyorin University, Tokyo, Japan) and maintained in high-glucose Dulbecco’s modified Eagle’s medium (DMEM) containing 10% calf serum and 1% penicillin-streptomycin at 37°C in a humidified atmosphere of 5% CO_2_. To induce the differentiation of the pre-adipocytes into mature adipocytes, 100% confluent cells were maintained for 2 days and changed to differentiation medium (DMEM containing 10% fetal bovine serum, 10 µg/ml insulin, 1 µM dexamethasone, 500 µM 3-isobutyl-1-methylxanthine, and 1 µM troglitazone). Two days later, the media were replaced with DMEM containing 10% fetal bovine serum and refreshed every other day for an additional 6 days. For the phosphate stimulation experiment, the cells were incubated in DMEM with phosphate buffer (Na_2_HPO_4_/NaH_2_PO_4_, pH 7.4) at day 8 of differentiation for 5 days. For the PTH and FGF23 stimulation experiments, mature adipocytes were cultured in DMEM supplemented with PTH or FGF23 for 5 days. The HepG2 human hepatocellular carcinoma cells were cultured in low-glucose DMEM. The medium was changed to low- or high-glucose DMEM with or without each experimental factor (phosphate, PTH, or FGF23) when the cells were 60% confluent, and the cells were incubated for a further 24 h.

### Real-time polymerase chain reaction analysis

 Total RNA was isolated from frozen animal tissues and cultured cells using RNAiso Plus (Takara Bio, Shiga, Japan). The quantity of the isolated RNA was determined using a NanoDrop spectrophotometer and cDNA was synthesized from the RNA template using M-MLV according to the standard method. Subsequently, SYBR Green-based quantitative real-time polymerase chain reaction (PCR) was performed using the synthesized cDNA and an appropriate primer set for each target gene with a StepOnePlus real-time PCR system (Applied Biosystems, Foster City, CA).

### Western blotting

Tissue was lysed in cold radioimmunoprecipitation buffer (25 mM Tris-HCl, pH 7.6, 150 mM NaCl, 1% NP-40, 1% sodium deoxycholate, and 0.1% sodium dodecyl sulfate) supplemented with a protease inhibitor and phosphatase inhibitor cocktail. For western blotting, 15 or 20 µg of protein was separated in a 10% polyacrylamide gel by sodium dodecyl sulfate–polyacrylamide gel electrophoresis and transferred onto a polyvinylidene difluoride membrane by electrophoresis. The membrane was blocked with 5% skim milk/Tris-buffered saline containing 0.02% Tween 20 for 1 h at room temperature and treated with each antibody: phosphorylated hormone-sensitive lipase (HSL) (Ser660), β-actin (Cell Signaling Technology, Beverly, MA), and HSL (Santa Cruz Biotechnology, Dallas, TX) overnight at 4°C. After washing with Tris-buffered saline containing 0.02% Tween 20, the membrane was incubated with an appropriate secondary antibody for 1 h at room temperature and visualized using a luminescent image analyzer (LAS-3000 UV mini, Fujifilm, Tokyo, Japan). The band was quantified using Multi Gauge software ver. 3.0 (Fujifilm).

### Histological analysis

The liver was fixed with 4% paraformaldehyde and embedded in paraffin. The tissue section was then cut into slices of 5 µm thickness, which were deparaffinized and stained with periodic acid–Schiff (PAS) and hematoxylin. The PAS-stained area of each tissue slice was measured using Image-Pro Plus ver. 5.1 (Media Cybernetics, Inc., Rockville, MD).

### Statistical analysis

Results were expressed as the mean ± SE. The significance of differences between the groups was determined by 1-way analysis of variance followed by the Tukey–Kramer post hoc test. The statistical calculations were performed using Excel Toukei 2006 (SSRI, Tokyo, Japan) and Prism ver. 5 (GraphPad Software, San Diego, CA). Differences with a *p* value <0.05 were considered statistically significant.

## Results

### High-phosphorus diet suppresses body fat accumulation

**Fig. 1:**
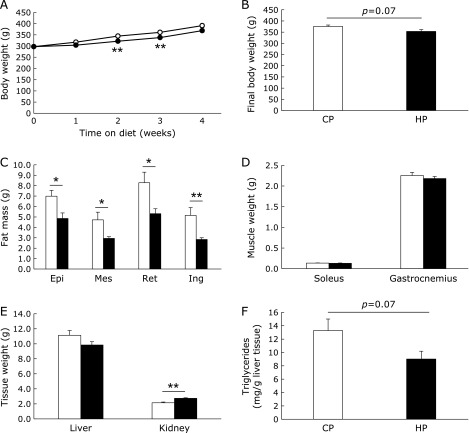
Body weight and tissue mass of high-phosphate diet-fed rats. Body weight changes during dietary intervention (A) and body weight at 4 weeks after 12 h fasting (B). The epidydimal (Epi), mesenteric (Mes), retroperitoneal (Ret), and inguinal (Ing) fat masses (C). Soleus, gastrocnemius (D), liver, and kidney (E) weights. Hepatic triglyceride levels (F). Open circle or column stands for control diet-fed group, closed circle or column for high-phosphate (HP) diet-fed group. Data are presented as mean ± SE. *n* = 5–6 rats per group. *p<0.05, **p<0.01.

**Fig. 2:**
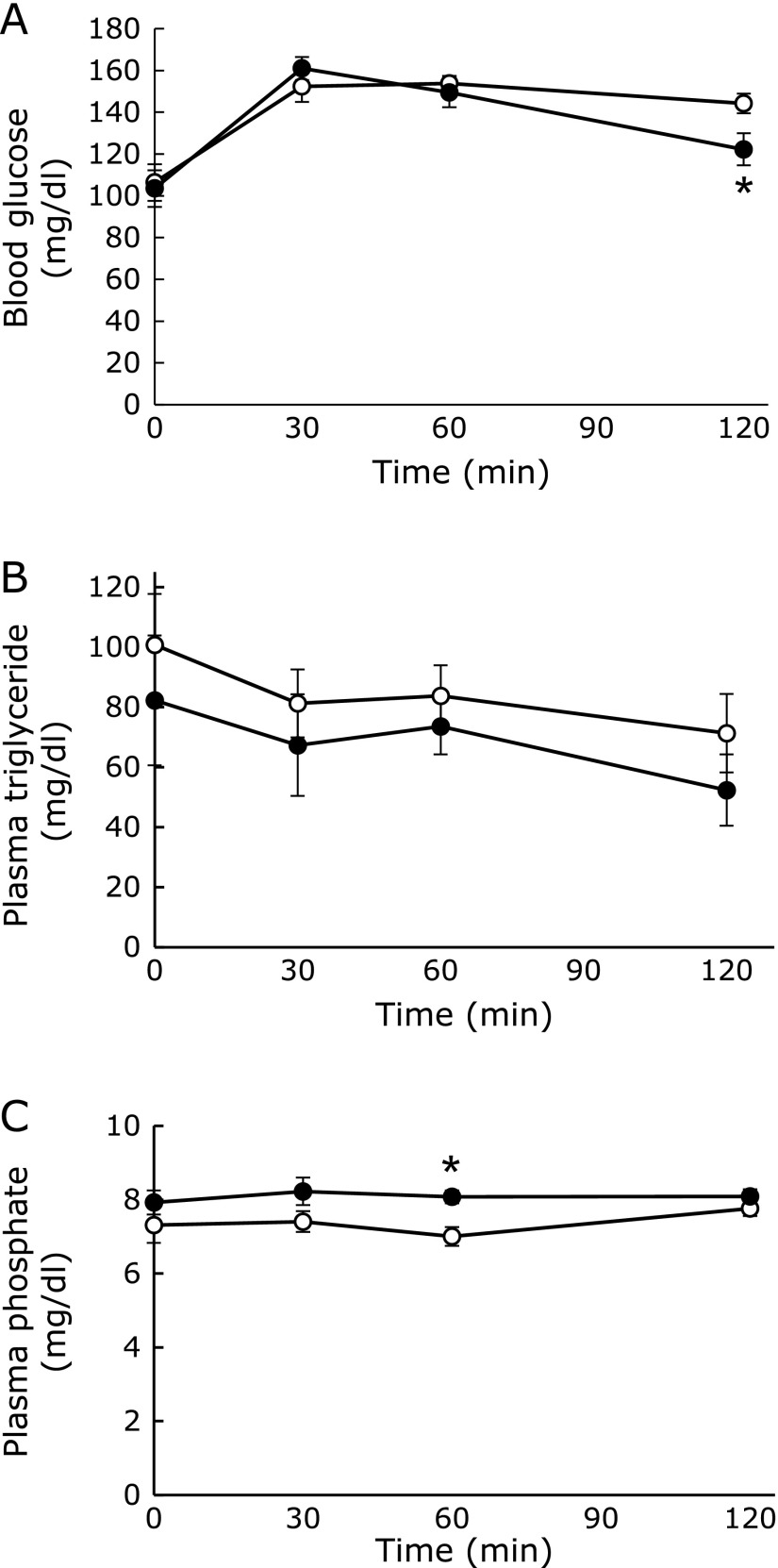
Nutrient absorption after the intake of a high-phosphate diet. Rats aged 8 weeks were fed either the control (open circle) or high-phosphate (HP) (closed circle) diet before the initiation of HP diet feeding. The serial changes of blood glucose (A), triglyceride (B), and phosphate (C) levels. Data are presented as mean ± SE. *n* = 5–6 rats per group. *p<0.05.

The amounts of food and energy intake did not significantly differ between the CP and HP diet-fed rats (Table 2). However, the body weight gain in the HP group was slightly smaller than that in the CP group (Fig. 1A and B). The HP group showed significantly lower visceral (epidydimal, mesenteric, and retroperitoneal) and subcutaneous (inguinal) fat masses than the CP group (Fig. 1C), while the weights of liver and skeletal muscle (soleus and gastrocnemius) did not significantly differ between the groups (Fig. 1D and E). In addition, the kidney weight of the HP group was significantly greater than that of the CP group (Fig. 1E). The hepatic triglyceride level in the HP group was slightly lower than that in the CP group (Fig. 1F). Those data suggested that the HP diet may suppress fat accumulation as previously reported.^(15)^ To exclude the possibility that this effect may be caused by the difference between the compositions of the CP and HP diets affecting the absorption rates of glucose and lipid, we tried to measure the postprandial increases in blood glucose and plasma triglyceride levels after a single feeding of each diet. The amounts of carbohydrate and lipid in both diets corresponded to 0.3 g/100 g body weight and 0.03 g/100 g body weight, respectively. No significant difference between the CP and HP groups was observed in the blood glucose levels at 0, 30, and 60 min after the feeding of each diet to the rats. However, at 120 min, the blood glucose levels in the HP group were lower than those of the CP group (Fig. 2A). By contrast, there was no difference between the CP and HP groups in the postprandial changes in plasma triglyceride levels (Fig. 2B). Postprandial plasma phosphate levels were significantly higher in the HP group at 60 min (Fig. 2C). In addition, we confirmed that the fecal weight and lipid content did not differ between the two diet groups (Table 2). These data suggest that there was no difference between the CP and HP groups in the intestinal absorption rates of glucose and lipid.

### Effects of dietary phosphate on serum and urinary biochemical data

The fasting plasma phosphate level in the HP group was slightly lower than that in the CP group, but the difference was not statistically significant (Table 3). By contrast, the concentrations of plasma PTH and FGF23 as well as urine phosphate were significantly higher in the HP group than in the CP group. There was no significant difference between the diet groups in the levels of plasma 1,25(OH)_2_D, calcium, and creatinine (Table 3). The levels of fasting blood glucose and plasma insulin as well as the HOMA-IR of the HP group were significantly lower than those of the CP group (Table 2). The levels of triglyceride, total cholesterol, and non-esterified fatty acid in plasma showed no significant differences between the two diet groups (Table 2).

### Effect of dietary phosphate on gene expression and lipase activation in white adipose tissue

**Fig. 3:**
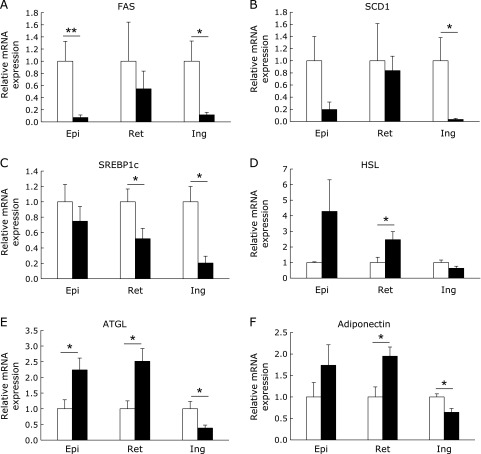
The lipogenic and lipolytic gene expression in three types of white adipose tissue. The mRNA expression of lipogenic and lipolytic genes in the epidydimal (Epi), retroperitoneal (Ret), and inguinal (Ing) white adipose tissues of rats after feeding the control (open column) or high-phosphate (HP) (closed column) diet for 4 weeks (A–F). The expression level of each target gene was normalized to that of the 36B4 housekeeping gene. Data are presented as mean ± SE. *n* = 5–6 rats per group. *p<0.05, **p<0.01.

**Fig. 4:**
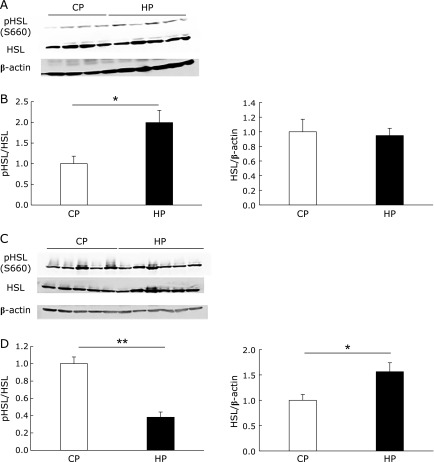
HSL phosphorylation in white adipose tissue. Immunoblotting using the p-HSL (Ser660), HSL, and β-actin antibodies in retroperitoneal (A and B) and epidydimal (C and D) white adipose tissues. The intensities of the luminescent signals were quantified using software (B and D). Data are presented as mean ± SE. *n* = 5–6 rats per group. *p<0.05, **p<0.001.

We next examined gene expression in three types of white adipose tissue to reveal the effects of phosphate on lipid metabolism. The mRNA expression of lipogenic enzymes such as fatty acid synthase (FAS) and stearoyl-CoA desaturase-1 (SCD1) was significantly or slightly lower in all the white adipose tissues of HP diet-fed rats, as compared with the control group (Fig. 3A and B). Sterol regulatory element-binding protein1c (SREBP1c) is a transcription factor for the FAS and SCD1 genes. The mRNA expression of SREBP1c was also lower in the white adipose tissues of the HP group (Fig. 3C). By contrast, the mRNA expression of the lipolytic enzymes HSL and adipose triglyceride lipase (ATGL), as well as that of adiponectin, which is an adipokine that enhances insulin sensitivity, were higher in the visceral (epidydimal and retroperitoneal) fat of the HP group, whereas their mRNA expression was lower in subcutaneous (inguinal) fat (Fig. 3D–F). We next investigated HSL phosphorylation in visceral fat, because HSL is known to be activated by phosphorylation at the residue serine 660.^(26)^ As shown in Fig. 4A and B, HSL phosphorylation in the retroperitoneal fat of the HP group was significantly higher than that of the CP group. Unexpectedly, the phosphorylation of HSL in the epidydimal fat of the HP group was significantly lower than that of the CP group. On the other hand, HSL protein expression was significantly increased in the HP group (Fig, 4C and D).

### Phosphate, PTH and FGF23 did not induce lipolytic gene expression in adipocyte

**Fig. 5:**
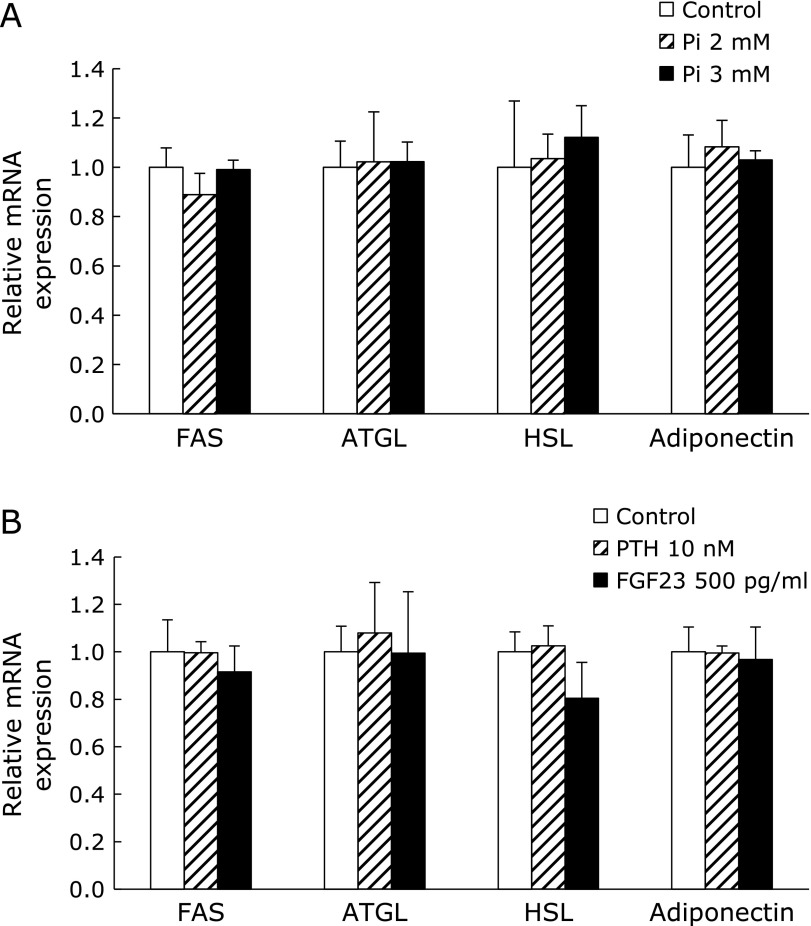
The effects of phosphate or phosphate regulators on 3T3-L1 cells. The expression of lipogenic and lipolytic genes in mature 3T3-L1 adipocytes cultured in medium supplemented with 2 or 3 mM phosphate (A), 10 nM PTH, or 500 pg/ml FGF23 (B) for 5 days. The control medium contained 0.9 mM phosphate. The expression level of each target gene was normalized to that of the housekeeping gene GAPDH. Data are presented as mean ± SE. *n* = 3 per group.

Although the fasting plasma phosphate level did not differ between the CP and HP groups, the random plasma phosphate level was significantly higher in the HP group than in the CP group (CP 4.88 ± 0.22 mg/dl, HP 8.43 ± 0.64 mg/dl) on the day before euthanization. Therefore, we investigated the direct effect of phosphate on adipocytes using 3T3-L1 cells. However, elevating the concentration of extracellular phosphate did not change the mRNA expression of FAS, ATGL, HSL, and adiponectin, unlike the result *in vivo* (Fig. 5A). Supplying exogenous PTH and FGF23 also had no effect on the expression of those genes (Fig. 5B).

### Effect of HP diet on the lipid metabolism in the liver

**Fig. 6:**
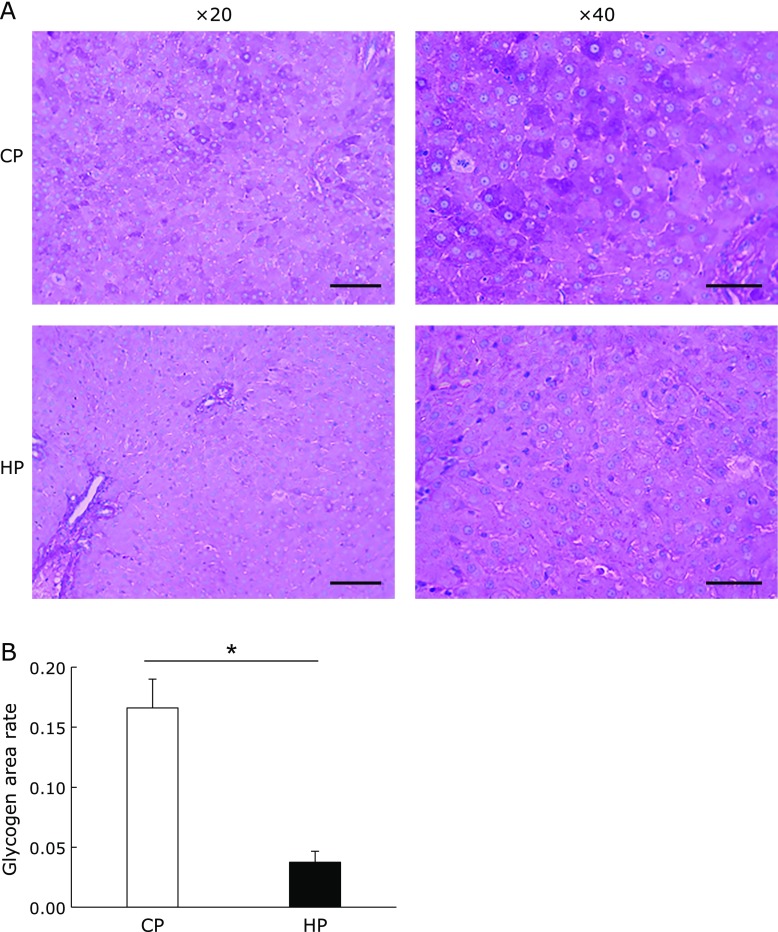
Hepatic glycogen and triglyceride levels. Representative liver histology by periodic acid–Schiff staining at 20× and 40× magnification (A). The scale bars represent 100 µm (left) and 60 µm (right). The stained area was quantified by software (B). Data are presented as mean ± SE. *n* = 5–6 rats per group. *p<0.01.

**Fig. 7:**
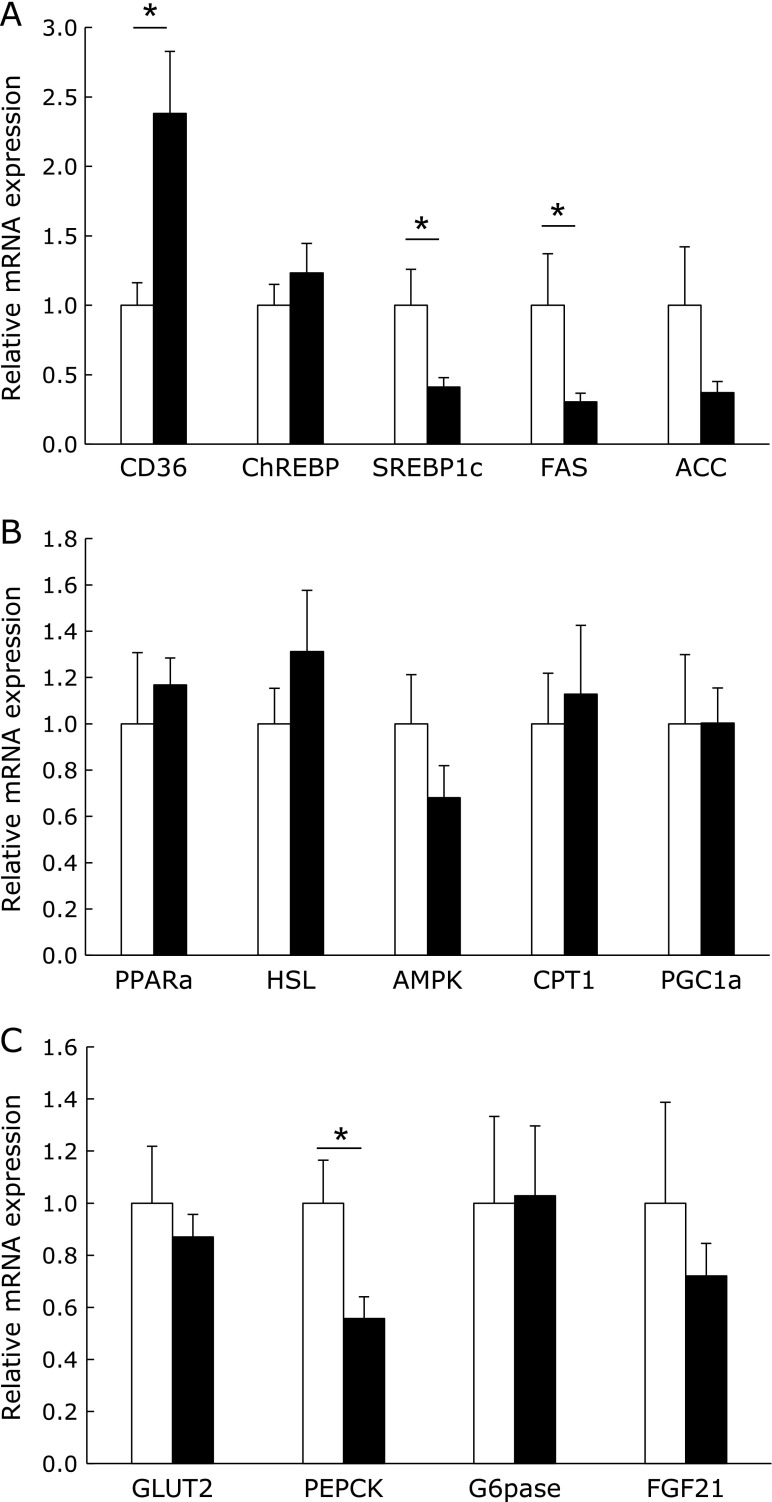
The gene expression in the liver. The mRNA expression in the liver of rats fed with the control (open column) or high-phosphate (HP) (closed column) diet for 4 weeks (A–C). The expression of each target gene was normalized to that of the β-actin gene. Data are presented as mean ± SE. *n* = 5–6 rats per group. *p<0.05.

The liver plays a central role in the systemic regulation of energy homeostasis though glycogenolysis, gluconeogenesis, and fat oxidation in response to a fasting state. We analyzed the amount of hepatic glycogen by the PAS staining of tissue sections. The amount of hepatic glycogen after 12 h of fasting was significantly lower in the HP group (Fig. 6A and B). In addition, the hepatic triglyceride level was slightly lower in the HP group, although the liver weight did not differ between the two diet groups. Therefore, we next investigated the expression of genes involved in lipid and glucose metabolism in the liver. The mRNA expression of fatty acid translocase (FAT/CD36) was significantly higher in the liver of the HP group rats; however, the expression of other lipogenic genes such as SREBP1c, FAS, and ACC expression was significantly or slightly lower (Fig. 7A). By contrast, the expression of lipolytic genes such as HSL, peroxisome proliferator-activated receptor (PPAR) α, AMP-activated protein kinase (AMPK), carnitine palmitoyltransferase 1 (CPT1), and PPARγ coactivator-1 (PGC1) α did not differ between the diet groups (Fig. 7B). The mRNA expression of phosphoenolpyruvate carboxykinase (PEPCK), which is the rate-limiting enzyme of gluconeogenesis, was significantly lower in the liver of the HP diet-fed rats, although the expression of other genes involved in glucose metabolism did not differ between the diet groups (Fig. 7C).

### PTH can induce lipogenic gene expression in HepG2 cells

**Fig. 8:**
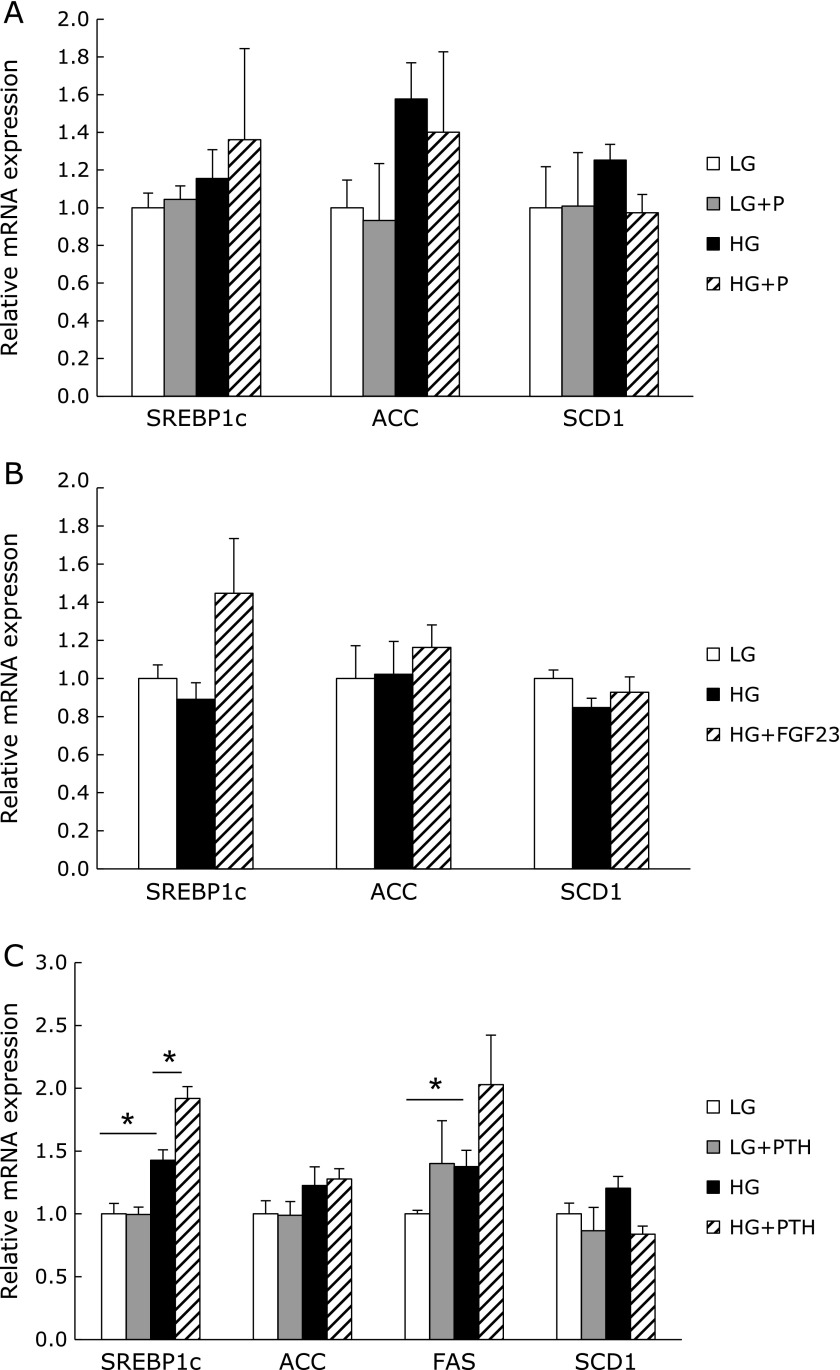
The effects of phosphate and phosphate regulators on HepG2 cells. The mRNA expression of HepG2 cells stimulated with 2 mM phosphate (A), 500 pg/ml FGF23 (B), or 10 nM PTH (C) for 24 h in low- or high-glucose medium. LG, low-glucose medium; HG, high-glucose medium. The expression of each target gene was normalized to that of the β-actin gene. Data are presented as mean ± SE. *n* = 3–4 per group. *p<0.05.

Our data suggested that the HP diet suppressed hepatic fat accumulation. In addition, the rats that were fed the HP diet showed increased PTH levels in the blood, and a previous study indicated that an elevated level of PTH in serum may be the predictive factor for nonalcoholic steatohepatitis in morbidly obese patients.^(27)^ Therefore, we next examined the effect of phosphate or PTH on hepatocytes using HepG2 cells. Since it is known that culture in a high-glucose medium can induce lipogenesis in HepG2 cells,^(28)^ we tried to culture the HepG2 cells in low- and high-glucose media supplemented with or without phosphate, PTH, or FGF23. The mRNA expression of lipogenic genes including SREBP1c, ACC, and SCD1 slightly increased when the cells were incubated in a high-glucose medium. High phosphate and FGF23 did not affect the expression of lipogenic genes (Fig. 8A and B). Although PTH induced lipogenic gene expression under the high-glucose condition, no induction of lipogenic gene expression was observed under the low-glucose condition (Fig. 8C).

## Discussion

The present study demonstrated that a high phosphate intake suppressed body fat accumulation by suppressing lipogenic gene expression and inducing lipolytic gene expression in visceral adipose tissue. In this study, the HP diet-fed rats showed a higher insulin sensitivity than the CP diet-fed rats. Insulin has been known to decrease plasma phosphate and glucose levels by promoting the transport of glucose and phosphate into cells, especially in skeletal muscle and adipose tissue. In addition, several studies have demonstrated that phosphate supplementation can promote insulin-mediated glucose uptake and decrease insulin secretion.^(29,30)^ Insulin also promotes glycogen synthesis and lipogenesis, while it suppresses lipolysis. The present study demonstrated that the accumulation of hepatic glycogen was lower in the HP group than in the CP group. Therefore, the suppression of body fat accumulation in the HP group is likely to be mediated by low plasma insulin levels. In addition, the HP group showed an increased mRNA expression of adiponectin in retroperitoneal white adipose tissue. Adiponectin is secreted from the white adipose tissue of lean individuals and can increase insulin sensitivity in various tissues including skeletal muscle and adipose tissues. The HP group showed lower levels of fasting blood glucose, insulin, hepatic glycogen, and HOMA-IR than the CP group. The low fat accumulation in the HP group is likely to have been mediated by a lower secretion of insulin, resulting in the suppression of lipogenesis and fat accumulation and the stimulation of lipolysis. Interestingly, there are some differences in gene expression and protein phosphorylation in response to the HP diet among different adipose tissues. For instance, the HP diet lowered ATGL and adiponectin gene expression in inguinal adipose tissue, and phosphorylation of HSL protein in epidydimal adipose tissue, inversely increased ATGL and adiponectin in retroperitoneal adipose tissue, and phosphorylation of HSL protein in epidydimal adipose tissue. Such differences in physiological response to a diet among different adipose tissues were reported in other previous studies,^(31,32)^ however, the reason has not been clarified.

Several previous studies have reported that markers of the metabolic syndrome such as body fat accumulation, the blood glucose level, and insulin resistance show both a negative correlation with the plasma phosphate level and a positive correlation with the concentration of PTH or FGF23.^(10,19,33)^ Therefore, we examined the possibility that phosphate, PTH, or FGF23 exert direct effects on adipose tissue or the liver through *in vitro* experiments using 3T3-L1 and HepG2 cells. However, none of those factors affected the gene expression in mature 3T3-L1 adipocytes after 5 days of treatment. A previous study showed that PTH can induce the suppression of glucose uptake through the insulin signaling pathway on 3T3-L1 cells.^(34)^ In addition, PTH can stimulate the activity of HSL through protein kinase A-dependent phosphorylation, resulting in lipolysis in 3T3-L1 cells.^(21)^ The present study demonstrated that the HP diet increased the phosphorylation of HSL in retroperitoneal fat. Therefore, the elevated level of PTH in HP diet-fed rats may partially mediate the suppression of fat accumulation. In HepG2 cells, PTH stimulated the gene expression of SREBP1c under the high-glucose condition, but not under the low-glucose condition. In addition, the supplementation of phosphate and FGF23 did not affect lipogenic gene expression. These data suggested that the elevation of extracellular phosphate, PTH, and FGF23 concentrations would not directly suppress hepatic lipogenesis.

A recent study demonstrated that the dietary supplementation of phosphate could be useful for the treatment of obesity.^(14)^ However, the long-term administration of a HP diet causes renal hypertrophy and fibrosis as well as vascular disorders such as medial calcification and endothelial dysfunction.^(35–37)^ Hyperphosphatemia is a common symptom in end-stage CKD patients. Such patients often show a low body weight and a hypercatabolic state known as protein-energy wasting.^(38)^ The results of the present study suggested that hyperphosphatemia or an excessive intake of phosphate in CKD patients may be a cause of protein-energy wasting. Several human and animal studies have demonstrated that higher plasma phosphate levels or a HP diet correlate with body weight reduction in CKD.^(39,40)^

In conclusion, we demonstrated that a high dietary intake of phosphate can suppress the accumulation of white adipose tissue by increasing lipolytic gene expression and decreasing lipogenic gene expression. These effects could be mediated by the lower level of fasting plasma insulin that occurred in response to the HP diet, but they were not caused by direct actions of plasma phosphate or phosphaturic hormones on adipose tissue or the liver.

## Figures and Tables

**Table 1:** The compositions of the experimental diet

	CP	HP
Composition (g)		
Casein	20	20
Cystine	0.3	0.3
beta-Corn starch	39.7	35
alpha-Corn starch	13.2	13.2
Sucrose	6.6	10
Soybean oil	7	7
Cellulose	5	5
Vitamin mix	1	1
Choline bitartrate	0.25	0.25
*tert*-Butylhydroquinone	0.0014	0.0014
Modified mineral mix	3.5	3.5
Calcium dihydrogen phosphate	0.2	3.7
Potassium dihydrogen phosphate	1.3	0.6
Sodium dihydrogen phosphate	0.4	0.3
Calcium carbonate	1.4	0
Total	100	100

Phosphate dose	0.6%	1.5%
Calcium dose	0.6%	0.6%

Energy (kcal/100 g)	393	377

CP, control phosphate diet; HP, high phosphate diet.

**Table 2:** Energy intake and biochemical data of glucose and lipid metabolism

	CP	HP	*p* value
Food intake (g/day)	17.9 ± 0.5	18.4 ± 0.6	NS
Energy intake (kcal/day)	70.47 ± 2.25	69.27 ± 2.10	NS
Fecal weight (g/day)	1.74 ± 0.23	1.76 ± 0.30	NS
Fecal triglyceride (mg/day)	0.94 ± 0.22	1.30 ± 0.11	NS
Blood glucose (mg/dl)	131.0 ± 5.5	110.8 ± 4.8	<0.05
Insulin (ng/ml)	6.56 ± 1.00	3.14 ± 0.47	<0.01
HOMA-IR	61.1 ± 9.3	24.4 ± 3.7	<0.01
Triglyceride (mg/dl)	96.4 ± 25.2	88.6 ± 22.3	NS
Total cholesterol (mg/dl)	75.0 ± 3.6	82.37 ± 9.1	NS
NEFA (mEq/dl)	0.72 ± 0.13	0.85 ± 0.12	NS

All data are presented as means ± SE. NEFA, nonesterified fatty acids. Blood sampling was performed at euthanization under fasting condition at 4 weeks after the experimental diet period was started (*n* = 5–6 for each group).

**Table 3:** Biochemical data of phosphate metabolism and renal function

	CP	HP	*p* value
Plasma			
Phosphate (mg/dl)	6.26 ± 0.51	4.93 ± 0.22	NS
Calcium (mg/dl)	8.05 ± 0.69	9.01 ± 1.41	NS
Creatinine (mg/dl)	0.53 ± 0.17	0.84 ± 0.16	NS
PTH (pg/ml)	94.19 ± 9.65	476.9 ± 135.2	<0.05
FGF23 (pg/ml)	257.0 ± 18.4	370.1 ± 40.0	<0.05
1,25(OH)_2_D (mg/dl)	256.8 ± 66.5	286.3 ± 30.2	NS
Urine			
Pi/Cre	0.65 ± 0.15	2.22 ± 0.23	<0.001
Ca/Cre	0.04 ± 0.02	0.05 ± 0.01	NS

All data are presented as means ± SE. PTH, parathyroid hormone; FGF23, fibroblast growth factor 23; 1,25(OH)_2_D, 1,25-dihydroxyvitamin D. Blood and urine sampling was performed at euthanization under fasting condition at 4 weeks after the experimental diet period was started (*n* = 5–6 for each group).
